# Impact of pharmacist-led chemotherapy counseling on health-related quality of life and psychological outcomes of oncology patients in cancer hospital: A single center, open-label, randomized controlled trial

**DOI:** 10.1016/j.rcsop.2025.100649

**Published:** 2025-08-26

**Authors:** Barsha Poudel, Sabina Sankhi, Nabin Pathak, Bijaya Basyal, Shishir Paudel, Nirmal Raj Marasine

**Affiliations:** a Department of Pharmacy, CiST College, Pokhara University, Baneshwor, Kathmandu, Nepal; b Department of Pharmacy, Shree College of Technology, Purbanchal University, Bharatpur, Chitwan, Nepal; c Department of Pharmacy and Clinical Pharmacology, Madan Bhandari Academy of Health Sciences, Hetauda, Bagmati Province, Nepal; d Drug Information Unit and Pharmacovigilance Cell, Department of Pharmacy, Hetauda Hospital, Madan Bhandari Academy of Health Sciences, Bagmati Province, Makwanpur, Hetauda, Nepal; e Pharmacy Unit, Prithvi Chandra Hospital, Ramgram-5, Nawalparasi, Nepal; f Department of Public Health, CiST College, Pokhara University, Baneshwor, Kathmandu, Nepal; g Kathmandu Institute of Child Health, Hepaliheight, Kathmandu, Nepal

**Keywords:** Chemotherapy counseling, Health-related quality of life, Oncology, Pharmacist, Randomized controlled trial

## Abstract

**Background:**

Chemotherapy often imposes significant psychological distress and impairs the health-related quality of life (HRQoL) of oncology patients. This study aimed to assess the impact of pharmacist-led chemotherapy counseling on HRQoL and psychological outcomes among oncology patients in a cancer hospital in Nepal.

**Methods:**

A single-blind, randomized controlled trial was conducted from December 2022 to July 2023 among 128 patients receiving chemotherapy. Patients were randomly allocated in a 1:1 ratio to a control group (usual care) or an intervention group (pharmacist-led counseling plus usual care). The intervention comprised a structured 20–25 min face-to-face counseling session and an educational leaflet addressing cancer, chemotherapy, psychological aspects, and lifestyle modifications. Primary outcomes—anxiety (GAD-7), depression (PHQ-9), and HRQoL (EQ-5D-3L)—were measured at baseline and three months post-intervention. For GAD-7 and PHQ-9, lower scores indicate fewer symptoms; for EQ-5D-3L, higher scores reflect poorer quality of life. Data were analyzed using an intention-to-treat approach and a generalized estimating equation (GEE) model.

**Results:**

The intervention group demonstrated significant improvements in anxiety (GAD-7: 13.57 ± 4.08 to 10.90 ± 3.79; *p* < 0.001), depression (PHQ-9: 17.71 ± 4.57 to 13.50 ± 4.17; p < 0.001), and HRQoL (EQ-5D-3L: 11.82 ± 3.41 to 9.85 ± 2.51; p < 0.001) at three months. The control group showed no significant changes in GAD-7 or EQ-5D-3L scores, but a small increase in PHQ-9 scores was observed. Adjusted GEE analyses confirmed significant reductions in anxiety and depression symptoms, as well as improvements in HRQoL for the intervention group compared with the control group.

**Conclusion:**

Pharmacist-led chemotherapy counseling significantly improved psychological well-being and HRQoL among oncology patients, highlighting the valuable role of pharmacists in comprehensive cancer care, especially in low-resource settings.

## 1 Introduction

Cancer is a major global health challenge and remains one of the leading causes of death and disability worldwide.^1^^,^^2^ In 2022, the World Health Organization (WHO) estimated 20 million new cancer cases and 9.7 million deaths globally, with numbers projected to exceed 35 million new cases by 2050.^3^ Beyond its high mortality, cancer imposes substantial physical, emotional, and social burdens, including prolonged treatment, adverse effects, frequent hospitalizations, and disruption of social and professional life, all of which negatively affect patients' psychological well-being and health -related quality of life (HRQoL).^4^

Chemotherapy, a key component of cancer treatment, is often administered alone or alongside surgery and/or radiotherapy.^5^ While effective, it is frequently associated with toxicities such as fatigue, loss of appetite, weight loss, alopecia, constipation, dyspnea, stomatitis, nausea, and fever, which are compounded by psychological consequences including depression, anxiety, and reduced self-esteem—factors known to impair HRQoL.^5^, ^6^, ^7^ Depression and anxiety occur in up to 20 % and 10 % chemotherapy patients, respectively,^8^ and can lead to poor treatment adherence, discontinuation of therapy, and suboptimal clinical outcomes.^6^ Moreover, psychological distress often intensifies during chemotherapy, may persist post-treatment, and is independently associated with increased mortality.^5^^,^^6^

Pharmacists could play a critical role in mitigating these challenges. Evidence shows that pharmacist-led chemotherapy counseling can reduce anxiety and depression, enhance self-esteem, and improve quality of life (QoL).^9^ Patients also value such counseling, highlighting its importance in oncology care.^10^ By improving patients' understanding of their disease, chemotherapy mechanisms, potential adverse effects, and self-management strategies, pharmacists help reduce both physical and psychological burdens, thereby supporting treatment adherence and enhancing overall QoL.^11^

Although many studies worldwide have demonstrated the benefits of pharmacist-led oncology interventions,^9^^,^^11^, ^12^, ^13^, ^14^, ^15^ evidence from low-resource settings such as Nepal remains scarce, largely due to limited pharmacist involvement in cancer care. This study addresses this gap by evaluating the impact of a structured pharmacist-led chemotherapy counseling session, supplemented with educational materials, on HRQoL and psychological outcomes among cancer patients receiving chemotherapy at a specialized cancer hospital in Nepal.

## 2 Methods

### 2.1 Study design and setting

A randomized, single-blind, parallel-group study was conducted from December 2022 to July 2023, with an allocation ratio of 1:1, to assess the effect of pharmacist-led chemotherapy counseling on HRQoL and psychological outcomes in oncology patients receiving chemotherapy at the National Hospital and Cancer Research Center (NHCRC), Lalitpur, Nepal. NHCRC is a premier healthcare institution dedicated to providing comprehensive cancer care, advanced medical treatments, and cutting-edge research. With a multidisciplinary team of specialists, it offers diagnostic, therapeutic, and palliative services to patients from across the country. The study was conducted and reported in accordance with the CONSORT 2010 checklist.^16^

### 2.2 Sample size

The sample size (*n* = 128) was determined using Rosner's formula for comparing two independent means, with 80 % power, an alpha level of 0.05, and a standardized effect size (Δ) of 0.5. This yielded 64 participants per group (control and intervention).

### 2.3 Recruitment and enrollment

Patients aged ≥18 years who were diagnosed with cancer, receiving chemotherapy at NHCRC, Lalitpur, and able to understand and provide written informed consent were included in the study. Those who did not meet the inclusion criteria or declined to participate were excluded prior to group allocation. A pharmacist confirmed patient eligibility through a face-to-face interview and a review of medical and medication records. Eligible participants were then contacted by the researcher and invited to attend a face-to-face meeting, during which the study's purpose was explained, written informed consent was obtained, and baseline assessments were conducted.

Following this, patients were allocated to the control group (CG) or the intervention group (IG) using simple random sampling in a 1:1 ratio with a random number generator. Randomization was performed using a computer-generated list of random numbers created in Microsoft Excel (version 2021). The control group was labeled 'C' and the intervention group 'I'. Each randomization code was placed in an opaque envelope labeled with a sequential number on the outside. These envelopes were prepared by individuals not involved in the trial to maintain allocation concealment. Once eligibility was confirmed, the next envelope in the sequence was opened to reveal the group assignment, which was then recorded in a randomization log to ensure an unbiased process.

Patients with limited literacy or those requiring assistance were supported by a study team member who clarified study procedures and helped complete the questionnaire when needed. Although blinding of participants and investigators was not possible, data entry and analysis were conducted by an independent team led by a statistician. Demographic, clinical, and treatment-related variables, including age, gender, marital status, ethnicity, education, occupation, diagnosis, cancer stage, and purpose of chemotherapy, were collected.

### 2.4 Control and intervention group

After random allocation, patients in the CG received usual care, which in our setting involved oncologist consultation, chemotherapy administration by nurses, and routine monitoring for side effects. Pharmacist involvement in the CG was limited to dispensing medications and answering brief treatment-related queries when requested, without providing structured pharmacist counseling. In contrast, patients in the IG received an educational intervention delivered by a pharmacist in a single, face-to-face structured session lasting 20–25 min and were provided with educational literature on the disease and chemotherapy, along with usual care. Patients also received an educational leaflet containing information on cancer, its diagnosis, treatment approaches, chemotherapy, mechanisms of action of chemotherapeutic drugs, potential side effects, psychological aspects of chemotherapy, and lifestyle modifications to support cancer management. Details of the pharmacist-administered intervention can be found in Supplementary File 1.

### 2.5 Outcomes

Changes in HRQoL and psychological outcomes were measured using the tools described in Table 1. These tools demonstrated good reliability in this study, with a Cronbach's alpha values of 0.81,0.84, and 0.83, respectively. The questionnaires used for the interviews were first translated from English to Nepali and then validated through a back-translation process to ensure accuracy. The Nepali version was pilot tested with 13 cancer patients who were not included in the final analysis. All interviews were conducted in the Nepali language.

**Table 1:** Tools used in assessing HRQoL, depression and anxiety level.

S.N.	Tools used	Used For	Descriptions	Scoring and directions
1	5-level European Quality of Life Scale (EQ-5D-3L)^17^	(HRQoL)	Measured QoL in the domains of mobility, self-care, usual activities, pain/discomfort and anxiety/depression.	Total score ranges from 5 to 15; lower scores indicate better quality of life.
2	Patient Health Questionnaire (PHQ-9)^18^	Depression level	9 items, each item scored as follows; not at all (0), several days (1), more than half the days (2) and nearly every day (3).	Total score ranges from 0 to 27; lower scores indicate fewer depressive symptoms
3	Generalized Anxiety Disorder (GAD-7)^19^	Anxiety level	Includes 7 items; each item is scored from 0 (not at all) to 3 (nearly every day)	Total score ranges from 0 to 21; lower scores indicate less anxiety.

The primary outcome of the study was the difference in mean scores of the GAD-7, PHQ-9, and EQ-5D-3L between the CG and IG three months after baseline.

### 2.6 Statistical analysis

Data were entered into EpiData version 3.1, and transferred to R (version 4.0.4) for analysis. The normality of the data was assessed using skewness, kurtosis, and the Shapiro–Wilk test. Descriptive statistics were used to summarize the data, while chi-square and Fisher's exact tests were applied to assess the distribution of variables between the CG and IG. The multiple imputation using chained equations (MICE) method was used to handle missing outcome data.

Study outcomes were analyzed following an intention-to-treat (ITT) approach, in accordance with the study protocol. Additionally, a paired *t*-test was performed to evaluate the impact of chemotherapy counseling on HRQoL and psychological outcomes. A generalized estimating equation (GEE) model was employed to account for the correlation of observations within clusters. Initially, an unadjusted model including only time and group variables was developed to estimate the unadjusted intervention effect. This model was subsequently refined by adjusting for baseline variables that differed significantly (*p* < 0.200) between the intervention and control groups, providing an adjusted estimate of the intervention effect. Results were reported as regression coefficients, with statistical significance set at *p* < 0.05.

### 2.7 Ethics

Ethical approval for this study was obtained from the Institutional Review Committee (IRC) of CiST College (IRC-CiST, Ref. No. 64/079/080), Naya Baneshwor, Kathmandu, Nepal. Written informed consent was obtained from all participants prior to the commencement of the study, and confidentiality was maintained throughout the research process.

## 3 Results

**Fig. 1:**
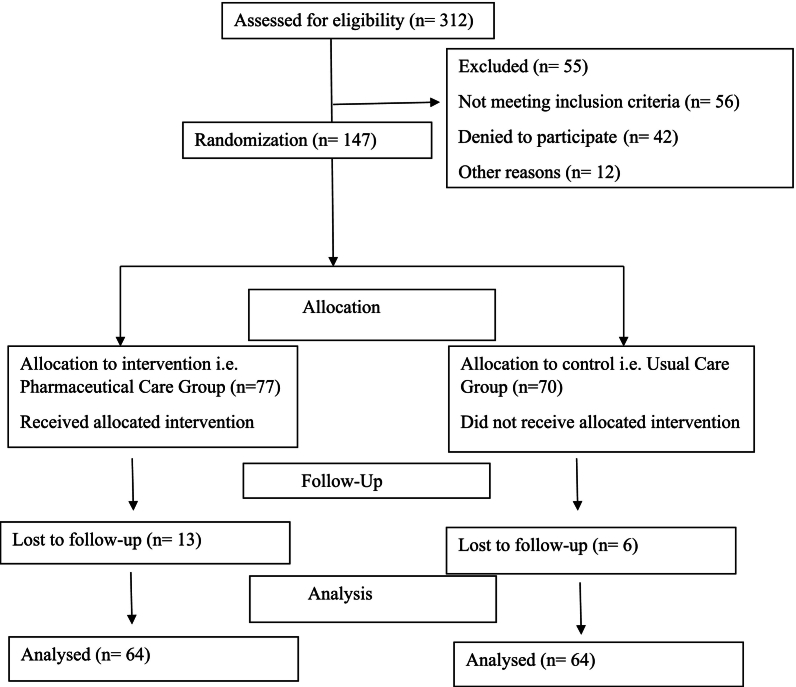
Participant enrollment, randomization, and inclusion in the final analysis.

A total of 312 patients were screened for eligibility, of whom 128 met the inclusion criteria. These patients were randomly assigned in a 1:1 ratio to the CG(*n* = 64) or the IG(n = 64) [Fig. 1]. No patients were lost to follow-up.

### 3.1 Socio-demographic and clinical characteristics

The mean age of patients was 57.36 ± 12.96 years in the CG and 52.05 ± 15.54 years in the IG. Females slightly outnumbered males (52.34 % vs. 47.66 %). Nearly one-third of participants (32.81 %) had attained primary-level education. Most were married (66.41 %), identified as Hindu (88.28 %), and were engaged in service-related occupations (28.13 %).

Regarding clinical characteristics, breast cancer were the major form of cancer (25.78%). Almost half of the patients (48.44 %) were at stage III, and adjuvant chemotherapy was the primary treatment purpose (59.38 %). Baseline characteristics are summarized in Table 2, with no statistically significant differences observed between groups.

**Table 2:** Baseline characteristics of study participants (*n* = 128).

Characteristics	Category	All patients, n (%)	CG, n (%)	IG, n (%)	*P*-Value
Age (years)	Mean ± SD		57.36 ± 12.957	52.05 ± 15.538	0.725
Gender	Male	61 (47.66)	27 (42.18)	34 (53.12)	0.215
	Female	67 (52.34)	37 (57.81)	30 (46.8)	
Education	Primary level	42 (32.81)	23 (35.93)	19 (29.68)	0.430
	Secondary level	30 (23.44)	14 (21.87)	16 (25)	
	Higher level	20 (15.63)	7 (10.93)	13 (20.31)	
	Illiterate	36 (28.13)	20 (31.25)	16 (25)	
Religion	Hindu	113 (88.28)	59 (92.18)	54 (84.3)	0.274
	Buddhist	8 (6.25)	2 (3.125)	6 (9.375)	
	Muslim	6 (4.69)	2 (3.125)	4 (6.25)	
	Christian	1 (0.78)	1 (1.56)	0 (0.00)	
Occupation	Business	29 (22.66)	15 (23.43)	14 (21.87)	0.637
	Service	36 (28.13)	19 (29.68)	17 (26.56)	
	Housemaker	32 (25.00)	18 (28.12)	14 (21.87)	
	Agriculture	28 (21.88)	11 (17.18)	17 (26.56)	
	Unemployed	3 (2.34)	1 (1.56)	2 (3.125)	
Marital status	Single	17 (13.28)	8 (12.5)	9 (14.06)	0.954
	Married	85 (66.41)	44 (68.75)	41 (64.06)	
	Widowed	24 (18.75)	11 (17.18)	13 (20.31)	
	Never married	2 (1.56)	1 (1.56)	1 (1.56)	
Diagnosis	Breast cancer	33 (25.78)	17 (26.56)	16 (25)	0.019
	Head and neck cancer	7 (5.47)	4 (6.25)	3 (4.68)	
	Stomach cancer	7 (5.47)	6 (9.375)	1 (1.56)	
	Ovarian cancer	15 (11.72)	9 (14.06)	6 (9.375)	
	Cervical cancer	5 (3.91)	0 (0.00)	5 (7.81)	
	Prostate cancer	7 (5.47)	1 (1.56)	6 (9.375)	
	Lymphoma	1 (0.78)	1 (1.56)	0 (0.00)	
	Lung cancer	25 (19.53)	14 (21.875)	11 (17.18)	
	Colon cancer	17 (13.28)	5 (7.81)	12 (18.75)	
	Gall bladder cancer	7 (5.47)	3 (4.68)	4 (6.25)	
	Others	4 (3.13)	4 (6.25)	0 (0.00)	
Stages of cancer	I	2 (1.56)	2 (3.125)	0 (0.00)	0.450
	II	39 (30.47)	19 (29.68)	20 (31.25)	
	III	62 (48.44)	29 (45.31)	33 (51.56)	
	IV	25 (19.53)	14 (21.87)	11 (17.18)	
Purpose of chemotherapy	Adjuvant	76 (59.38)	38 (59.37)	38 (59.37)	0.999
	Non adjuvant	52 (40.63)	26 (40.62)	26 (40.62)	

#others; skin cancer, leukemia, thyroid cancer.

Within group, changes in the primary outcomes from baseline to the 3-months follow-up are presented in Table 3. In the intervention group, mean GAD-7 scores decreased significantly from 13.57 ± 4.08 at baseline to 10.90 ± 3.79 at follow-up (*p* < 0.001), and mean PHQ-9 scores declined from 17.71 ± 4.57 at baseline to 13.50 ± 4.17 at follow-up; *p* = 0.901), indicating notable improvements in anxiety and depressive symptoms. The intervention group also demonstrated a significant improvement in mean EQ-5D-3L scores, from11.82 ± 3.41at baseline to 9.85 ± 2.51 at follow-up (*p* < 0.001), reflecting enhanced HRQoL. In contrast, the control group showed no significant changes in GAD-7 scores (baseline: 12.53 ± 5.45 vs. follow-up: 12.54 ± 5.45; *p* = 0.901) and EQ-5D-3L scores (baseline: 11.31 ± 3.17 vs. follow-up: 11.04 ± 3.23; *p* = 0.148). Interestingly, the control group showed a small but statistically significant increase in PHQ-9 scores, rising from 16.68 ± 5.19 at baseline to 17.04 ± 5.01 at follow-up (*p* < 0.001).

**Table 3:** Changes in HRQoL and psychological outcomes between intervention and control groups.

Outcome measures	Differences of HRQoL and Psychological outcomes at 95 % confidence interval								Between- group mean differences
	Intervention group				Control group				
	Baseline Mean ± SD	Follow-up Mean ± SD	Mean Difference (*E*- B)	p-within group	Baseline Mean ± SD	Follow-up Mean ± SD	Mean Difference (*E*-B)	p-within group	p-value
GAD-7	13.57 ± 4.08	10.90 ± 3.79	−2.67	<0.001	12.53 ± 5.45	12.54 ± 5.45	0.01	0.901	<0.001
PHQ-9	17.71 ± 4.57	13.50 ± 4.17	−4.21	<0.001	16.68 ± 5.19	17.04 ± 5.01	0.36	<0.001	<0.001
EQ-5D-3L	11.82 ± 3.41	9.85 ± 2.51	−1.97	<0.001	11.31 ± 3.17	11.04 ± 3.23	−0.27	0.148	<0.001

GAD-7: Generalized Anxiety Disorder-7; PHQ-9: Patient Health Questionnaire-9: EQ-5D-3L: EuroQol 5-Dimensions 3-Levels: HRQol: Health Related Quality of Life; SD: Standard deviation. Statistically significant at p < 0.01.

The effect of the intervention on primary outcomes, analyzed using a GEE model, is summarized in Table 4. In the adjusted model, after controlling for baseline variables with *p* < 0.200, the intervention group showed a statistically significant reduction in anxiety symptoms (GAD-7; β₁ = −0.19; 95 % CI: −0.399 to −0.127; *p* < 0.001) and depressive symptoms (PHQ-9; β₁ = −0.29 (95 % CI: −0.388 to −0.153; p < 0.001) compared with the control group over the 3-month follow-up. Furthermore, the intervention was associated with a significant improvement in HRQoL (EQ-5D-3L; β₁ = −0.12; 95 % CI: 0.100 to 0.09; *p* = 0.001) in the adjusted model.

**Table 4:** The effect of intervention on HRQoL and psychological outcomes using Generalized Estimating Equations (GEE).

Outcome measures	Intervention effects			
	Unadjusted regression coefficient, β₀ (95 % CI)	p- value	Adjusted regression coefficient, β_1_ (95 % CI)	p-value
GAD-7	−0.17 (−0.377 to −0.096)	0.001	−0.19 (−0.399 to −0.127)	<0.001
PHQ-9	−0.24 (−0.372 to −0.128)	<0.001	−0.29 (−0.388 to −0.153)	<0.001
EQ-5D-3L	−0.10 (−0.113 to 0.088)	0.021	−0.12 (0.100 to 0.09)	0.001

GAD-7: Generalized Anxiety Disorder-7; PHQ-9: Patient Health Questionnaire-9: EQ-5D-3L: EuroQol 5-Dimensions 3-Levels: HRQoL: Health Related Quality of Life; SD: Standard deviation.

Adjusted for age, gender, religion, education, occupation, marital status, diagnosis, stage of cancer, and purpose of therapy as these variables were statistically significant at p < 0.20 in the univariate analysis. Statistically significant at p -value < 0.05.

## 4 Discussion

Cancer diagnosis and treatment, particularly chemotherapy, impose substantial physical and psychological burdens on patients, notably anxiety and depression, which can markedly reduce quality of life. This study evaluated the impact of pharmacist-led chemotherapy counseling on psychological outcomes and HRQoL among cancer patients in a specialized cancer hospital in Nepal. The intervention group demonstrated significant improvements in anxiety, depression, and health- related quality of life at three months compared with the control group, underscoring the important role of pharmacists in supportive oncology care, especially in resource-limited settings.

Depression and anxiety are among the most prevalent psychological challenges in cancer patients, occurring at diagnosis, during treatment, and in survivorship when coping with the fear of recurrence.^20^ These conditions are linked to poor clinical outcomes, including increased mortality, inadequate pain management, reduced treatment adherence, and decreased engagement in long-term therapy.^8^^,^^9^ In this study, the marked reduction in depression and anxiety symptoms in the intervention group suggests that pharmacist-led education may improve understanding of the disease trajectory, make chemotherapy more comprehensible, build confidence in managing side effects, and alleviate psychological distress.^12^^,^^21^ These findings are consistent with systematic reviews reporting that pharmacist-led interventions reduce psychological burden and improve quality of life in oncology patients.^13^^,^^14^

Quality of life is a major concern in cancer care, as chemotherapy often disrupts physical, emotional, and social well-being. Beyond medical treatment, patients benefit from comprehensive support and counseling to help them cope.^9^^,^^15^^,^^22^ In the present study, HRQoL improved significantly in the intervention group but not in controls, likely due to the pharmacists ability to address informational and emotional needs, enabling better symptom management and adaptation to treatment challenges.^15^ Similar improvements have been documented in other pharmacist-led educational interventions targeting cancer patients.^9^^,^^11^^,^^22^^,^^23^

Interestingly, the control group showed no change in anxiety or HRQol but experienced a small, statistically significant increase in depressive symptoms. This underscores the heightened psychological vulnerability of patients lacking structured emotional and educational support during chemotherapy and reinforces the pharmacists role within the multidisciplinary cancer care team.

These findings are especially relevant to low- and middle-income countries (LMICs) like Nepal, where patients often face substantial psychological challenges due to limited mental health services and inadequate patient education. Integrating pharmacists into oncology teams can bridge these gaps by enhancing education, encouraging active participation in therapy, and providing psychological support—particularly where oncology services are under-resourced.^11^^,^^12^^,^^24^

Similar studies in other LMICs, notably Malaysia, have shown that pharmacist-led chemotherapy counseling can significantly improve quality of life and reduce anxiety and depression.^4^^,^^5^^,^^9^ The findings are consistent with these results, despite contextual differences: in Malaysia, pharmacists are routinely integrated into oncology care, and interventions often involve multiple sessions, whereas this study used a single 20–25 min session in a setting with limited pharmacist involvement. The positive outcomes observed in this resource-limited context highlight the feasibility and scalability of such interventions in similar LMICs. A recent systematic review supports the effectiveness of pharmacist-led oncology interventions in LMICs, although the intervention formats and outcome measures vary.^13^

Although conducted in a low-resource setting, these findings may also be relevant to rural and regional areas of high-income countries, where patients face comparable challenges, including limited access to oncology specialists, scarce mental health services, and inadequate patient education. In such contexts, pharmacist-led counseling could similarly help bridge gaps in care, optimize treatment adherence, and improve both psychological well-being and quality of life.

To the best of our knowledge, this is the first study in Nepal to evaluate the impact of pharmacist-led chemotherapy counseling on psychological outcomes and HRQoL among oncology patients. The single-blind randomized controlled trial design enhances the validity of the findings. The brief intervention (20–25 min) demonstrates feasibility, scalability, and cost-effectiveness in resource-limited cancer care settings. The use of a standardized educational leaflet addressing chemotherapy and lifestyle modifications further aligns with best practices in patient-centered care.

Despite the significant improvements observed in primary outcomes, several limitations should be acknowledged. First, the single-center, open-label design may limit the generalizability of our findings and introduce potential bias. As both participants and study staff were aware of group allocation, there is a possibility that this awareness influenced patients responses to questionnaires or the way data were collected, potentially leading to an over- or underestimation of the intervention's effect.

Second, the relatively short three-month follow-up period may not have been sufficient to capture long-term effects of the intervention, such as the sustained improvement or relapses in depression and anxiety symptoms, maintenance of HRQoL, and potential downstream impacts on treatment adherence. Third, cultural factors that could influence symptom perception and reporting were not explicitly addressed. Future studies should consider employing cluster randomized designs to minimize contamination and bias, extending follow-up, durations to assess sustainability, incorporating cost-effectiveness analyses, and conducting multi-center trials across diverse populations to enhance the robustness and applicability of evidence supporting pharmacist-led oncology interventions.

## 5 Conclusion

Pharmacist-led chemotherapy counseling significantly improved psychological well-being and HRQoL among cancer patients undergoing chemotherapy. These findings highlight the vital role of pharmacists within multidisciplinary oncology teams, especially in resource-limited settings. Future multi-center studies with extended follow-up periods and cost-effectiveness analyses are needed to validate these results and guide the integration of pharmacist-led supportive care into evidence-based healthcare policies.

The following are the supplementary data related to this article.

**Supplementary Fig. S1:**
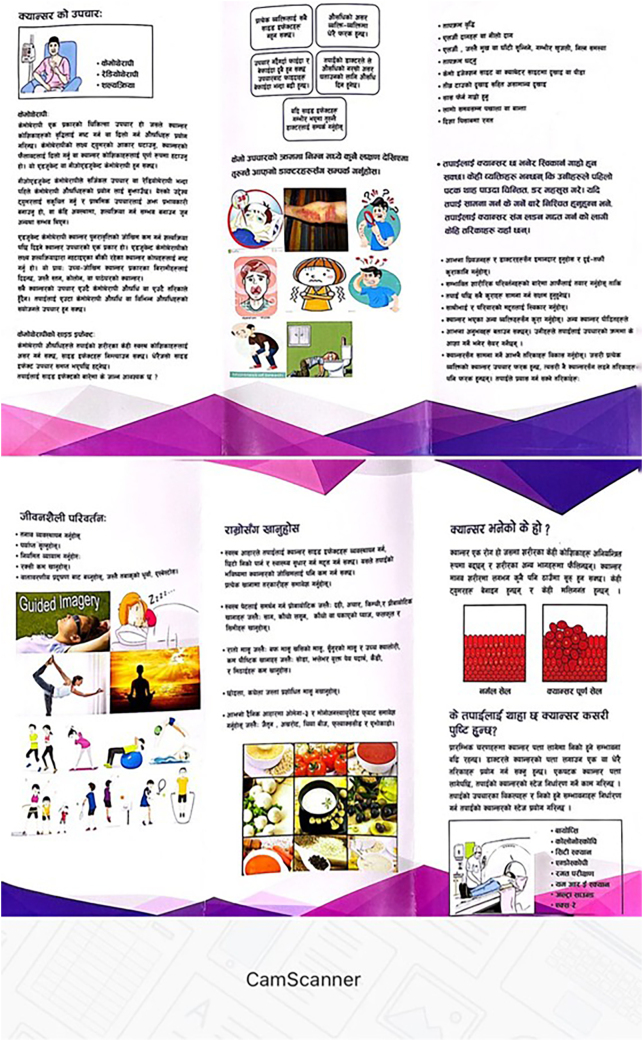

Supplementary material 1 Supplementary material 1

Supplementary material 2 Supplementary material 2

## Availability of data and materials

Data sets and materials for information in this manuscript can be provided by the corresponding author upon reasonable request.

## CRediT authorship contribution statement

**Barsha Poudel:** Resources, Project administration, Data curation, Conceptualization. **Sabina Sankhi:** Writing – review & editing, Writing – original draft, Validation, Methodology, Formal analysis. **Nabin Pathak:** Writing – review & editing. **Bijaya Basyal:** Writing – review & editing. **Shishir Paudel:** Writing – review & editing, Methodology. **Nirmal Raj Marasine:** Writing – review & editing, Writing – original draft, Visualization, Validation, Supervision, Software, Resources, Project administration, Methodology, Investigation, Formal analysis, Conceptualization.

## Declaration of competing interest

The authors declare that they have no known competing financial interests or personal relationships that could have appeared to influence the work reported in this paper.
