# Supplementary material for: Impact of pharmacist-led chemotherapy counseling on health-related quality of life and psychological outcomes of oncology patients in cancer hospital: A single center, open-label, randomized controlled trial
Source: Explor Res Clin Soc Pharm. 2025 Aug 26;20:100649. doi: 10.1016/j.rcsop.2025.100649 (PMC12444174; doi:10.1016/j.rcsop.2025.100649)
Supplement: Supplementary material 1 [file mmc2.docx]

# **APPENDIXES**

Annex 1: Socio-demographic information of the participants

| Characteristics | Options |
| --- | --- |
| Age |  |
| Gender | Male Female Others |
| Religion | Hindu Buddhist Muslim Christian Others |
| Education | Primary level Secondary level Higher level Illiterate |
| Occupation | Business Service Housemaker Agriculture Unemployed |
| Marital status | Single Married Widowed Never married |
| Cancer Diagnosis | Breast cancer Head and neck cancer Stomach cancer Ovarian cancer Cervical cancer Prostrate cancer Leukemia Lymphoma |
| Stages of cancer | I II III IV |
| Purpose of chemotherapy | Adjuvant Neo adjuvant |

**Annex 2: Generalized Anxiety Disorder 7-item (GAD-7)**

Generalized Anxiety Disorder 7-item (GAD-7) is a easy to perform initial screening tool for generalized anxiety disorder.

| **S.N** | **Over the last 2 weeks, how often have you been bothered by the following problems?** | **Not at all**  **0** | **Several days**  **+1** | **More than half the day**  **+2** | **Nearly every day**  **+3** |
| --- | --- | --- | --- | --- | --- |
| 1. | Feeling nervous, anxious or on edge |  |  |  |  |
| 2. | Not being able to stop or control worrying |  |  |  |  |
| 3. | Worrying too much about different things |  |  |  |  |
| 4. | Trouble relaxing |  |  |  |  |
| 5. | Being so restless that it is hard to sit still |  |  |  |  |
| 6. | Becoming easily annoyed or irritable |  |  |  |  |
| 7. | Feeling afraid as if something awful might happen |  |  |  |  |

Annex 3: patient health questionnaire-9 (PHQ-9)

The PHQ-9 is a multipurpose instrument for screening and diagnosing. Monitoring and measuring the severity of depression.

| **S.N** | **Over the last 2 weeks, how often have you been bothered by the following problems?** | **Not at all**  **0** | **Several days**  **+1** | **More than half the days**  **+2** | **Nearly every day**  **+3** |
| --- | --- | --- | --- | --- | --- |
| 1. | Little interest or pleasure in doing things |  |  |  |  |
| 2. | Feeling down, depressed or hopeless |  |  |  |  |
| 3. | Trouble falling asleep, staying asleep, or sleeping too much |  |  |  |  |
| 4. | Feeling tired or having little energy |  |  |  |  |
| 5. | Poor appetite or overeating |  |  |  |  |
| 6. | Feeling bad about yourself - or that you're a failure or have let yourself or your family down |  |  |  |  |
| 7. | Trouble concentrating on things, such as reading the newspaper or watching television |  |  |  |  |
| 8. | Moving or speaking so slowly that other people could have noticed. Or, the opposite - being so fidgety or restless that you have been moving around a lot more than usual |  |  |  |  |
| 9. | Thoughts that you would be better off dead or hurting yourself in some way |  |  |  |  |

Annex 4: EQ-5D-3L

EQ-5D-3L is a tool for evaluating quality of life.

By placing a tick in one box in each group, please indicate which statements best describe your health today.

**Mobility**

I have no problems walking about

I have some problems in walking about

I am confined to bed

**Self-Care**

I have no problems with self-care

I have some problems washing or dressing myself

I am unable to wash or dress myself

**Usual Activities** (e.g, work, study, housework, family or leisure activites)

I have no problems with performing my usual activities

I have some problems with performing my usual activities

I am unable to perform my usual activities

**Pain/Discomfort**

I have no pain or discomfort

I have moderate pain or discomfort

I have extreme pain or discomfort

**Anxiety/Depression**

I am not anxious or depressed

I am moderately anxious or depressed

I am extremely anxious or depressed
