# Supplementary material for: Impact of pharmacist-led chemotherapy counseling on health-related quality of life and psychological outcomes of oncology patients in cancer hospital: A single center, open-label, randomized controlled trial
Source: Explor Res Clin Soc Pharm. 2025 Aug 26;20:100649. doi: 10.1016/j.rcsop.2025.100649 (PMC12444174; doi:10.1016/j.rcsop.2025.100649)
Supplement: Supplementary material 2 [file mmc3.docx]

**What is cancer?**

Cancer is a disease in which some of the body’s cells grow uncontrollably and spread to other parts of the body. Cancer can start almost anywhere in the human body. Some tumors are benign and some are malignant.


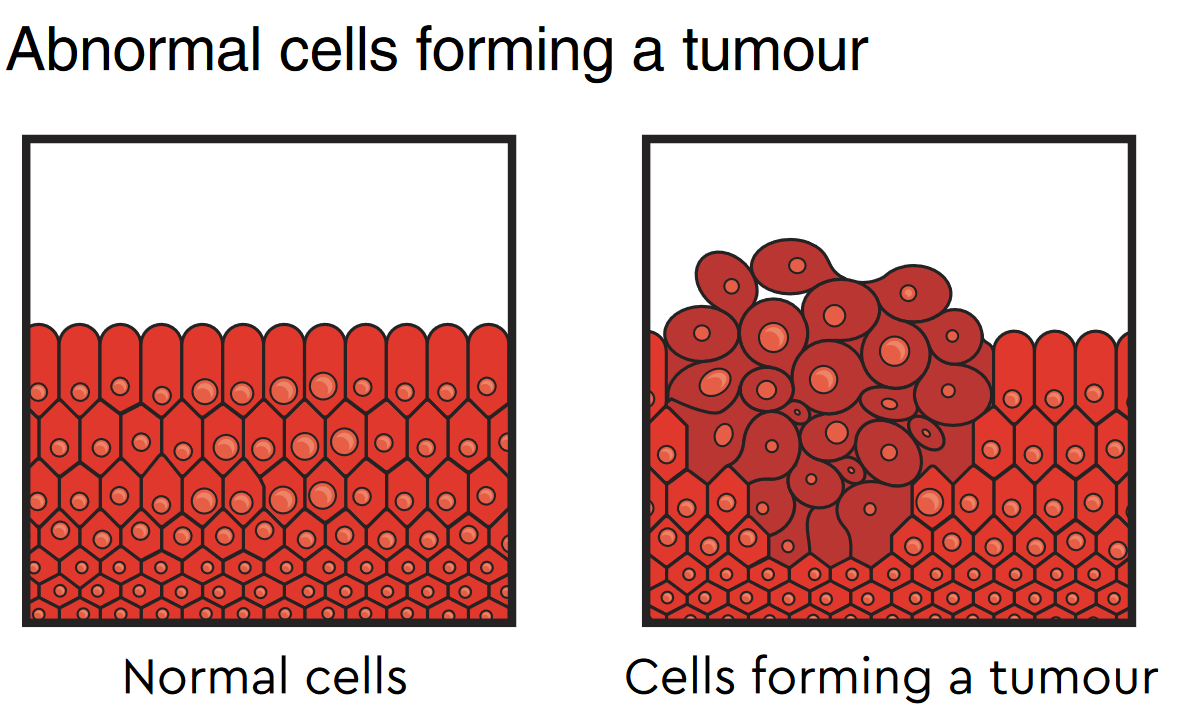


Do you know what confirms cancer?

Diagnosing cancer at its earliest stages often provides the best chance for a cure. Your doctor may use one or more approaches to diagnose cancer. Once cancer is diagnosed, your doctor will work to determine the extent (stage) of your cancer. Your doctor uses your cancer's stage to determine your treatment options and your chances for a cure.


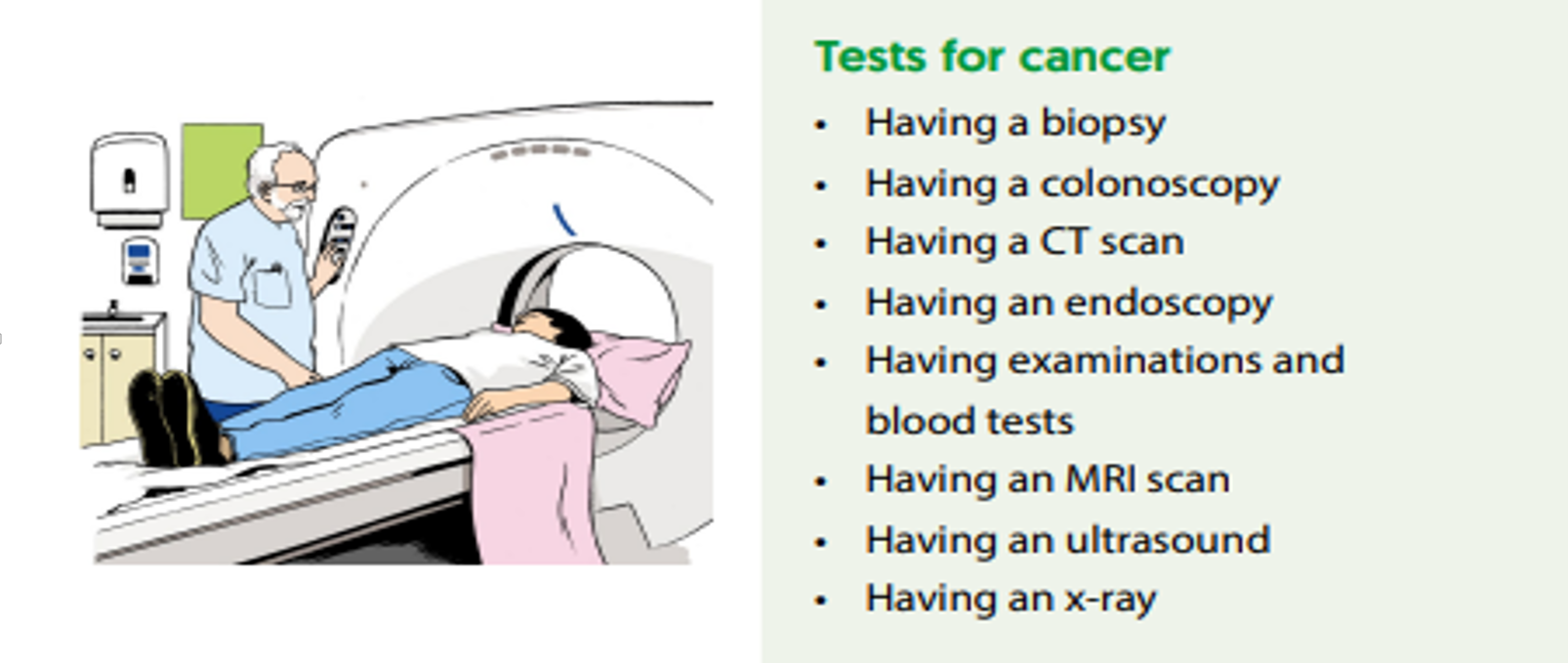


**Treatment of cancer:**


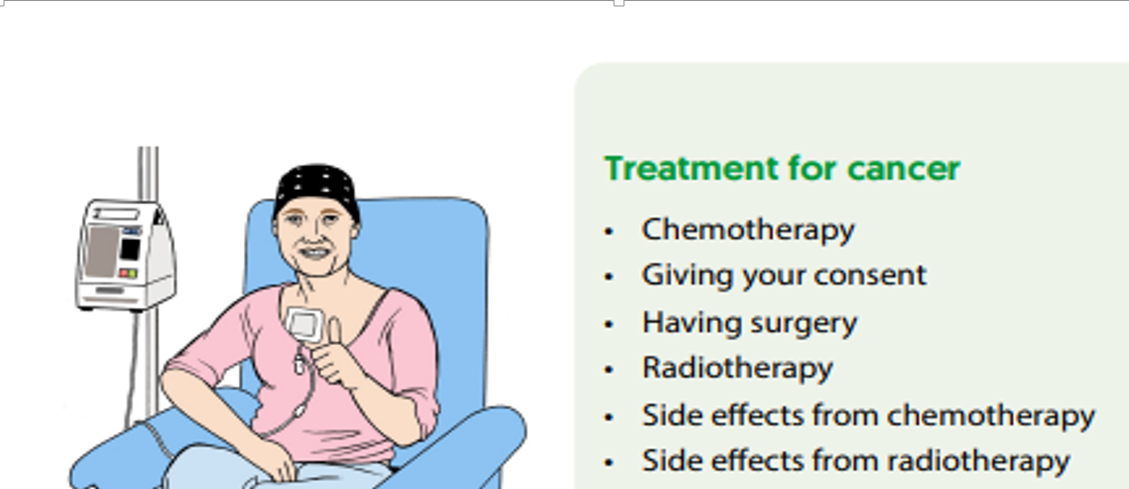


**Chemotherapy:**

Chemotherapy is a type of medical treatment that uses drugs to destroy or slow the growth of cancer cells. It is often used as part of a comprehensive treatment plan for cancer, which may also include surgery, radiation therapy, or immunotherapy. The goal of chemotherapy is to shrink the size of the tumor, slow the spread of the cancer, or eliminate the cancer cells altogether. It can be adjuvant or neoadjuvant chemotherapy.

**Neoadjuvant chemotherapy** refers to the administration of chemotherapy drugs prior to surgical treatment or radiation therapy. The purpose is to shrink the tumor and make the primary treatment more effective, or in some cases, make it possible to perform surgery that would not have been possible otherwise.

**Adjuvant chemotherapy** is a type of cancer treatment given after surgery to reduce the risk of cancer recurrence. The goal of adjuvant chemotherapy is to destroy any remaining cancer cells that may not have been removed by surgery. It is often given to patients with high-risk cancer types, such as breast, colon, or ovarian cancer.

Chemotherapy is usually given as several sessions of treatment, with rest periods in between each session. The rest period allows your body to recover from any side effects. It also allows the number of healthy cells in your blood to go back to normal. Chemotherapy and the rest period make up a cycle of your treatment.

**Side effects of chemotherapy:**

Chemotherapy drugs can affect some of the healthy cells in your body, causing side effects. Most side effects will go away after treatment finishes.

**What do I need to know about side effects?**

Side effects are unavoidable and benefits from the treatment is higher than the risk

Every person doesn’t get every side effect, and some people get few, if any.

The severity of side effects (how bad they are) varies greatly from person to person.

Your healthcare provider may give you medicines to help prevent certain side effects before they happen.

If side effects become severe immediately contact to the healthcare provider

Some chemo drugs cause long-term side effects, like heart or nerve damage or fertility problems. Still, many people have no long-term problems from chemo.

Contact your cancer care team right away if you have any of the following symptoms during chemo treatment:


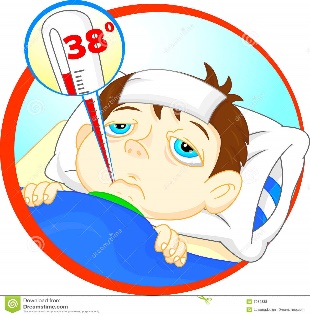

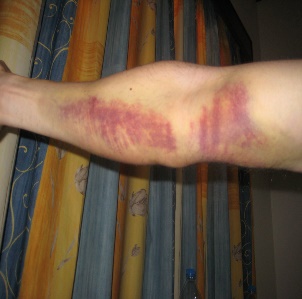

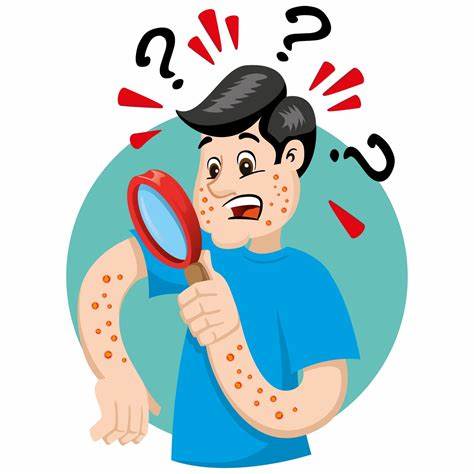

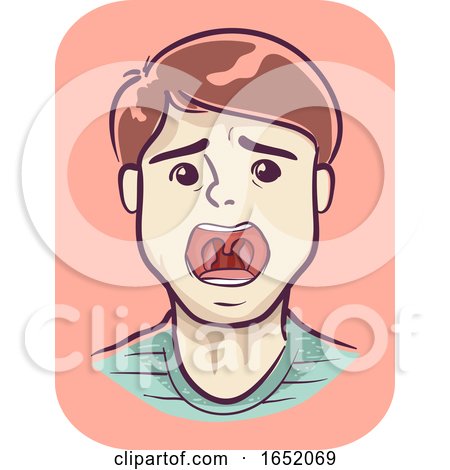


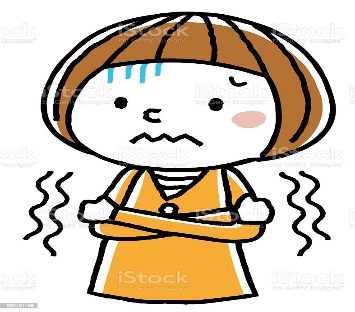

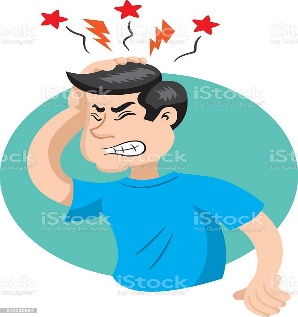

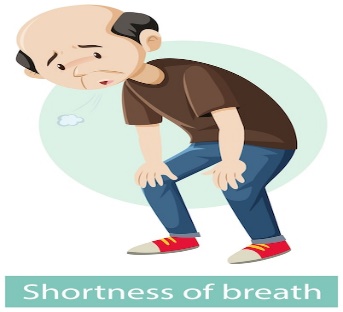

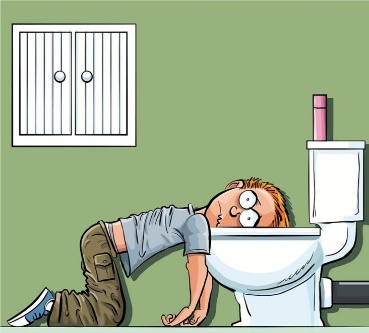


- A fever higher than what your cancer care team has instructed (usually 100.5°F -101°F or greater (taken by mouth)
- Bleeding or unexplained bruising
- A rash
- An allergic reaction, such as swelling of the mouth or throat, severe itching, trouble swallowing
- Intense chills
- Pain or soreness at the chemo injection site or catheter site
- Unusual pain, including intense headaches
- Shortness of breath or trouble breathing (If you’re having trouble breathing call 911 first.)
- Long-lasting diarrhea or vomiting
- Bloody stool or blood in your urine.

**Learning that you have cancer can be hard. Some people say they felt anxious, afraid or overwhelmed when they were first diagnosed. If you aren't sure what to do to cope, here are some ideas to help you deal with a cancer diagnosis.**

- Have honest, two-way communication with your loved ones, health care providers and others.
- Prepare yourself now about possible physical changes so that you'll be able to deal with everything later.
- Let friends and family help you
- Talk to other people with cancer. Other cancer survivors can share their experiences. They can tell you what to expect during treatment.
- Develop your own ways to deal with cancer. Just as each person's cancer treatment is different, so are the ways of dealing with cancer. Ideas you can try:

**Lifestyle Changes to Improve Your Cancer Care**

- Manage stress
- Get enough sleep
- Exercise regularly
- [Limit alcohol.](https://www.cancer.net/node/24981)
- Avoid environmental toxins


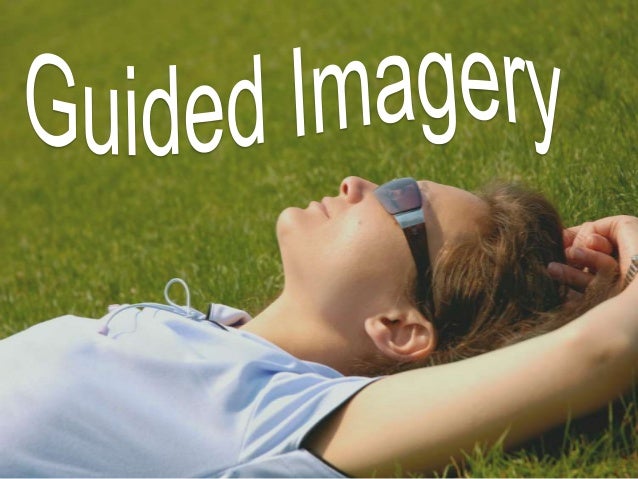

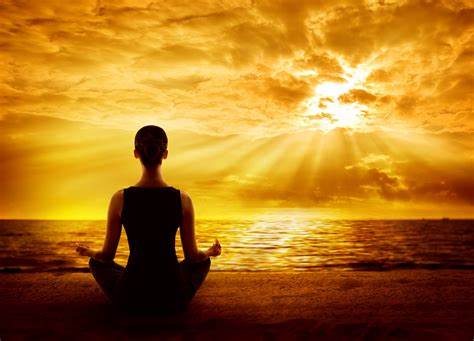

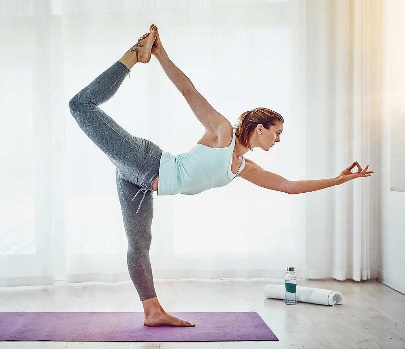


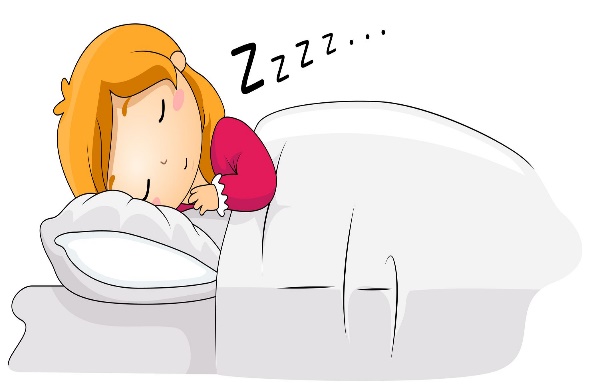

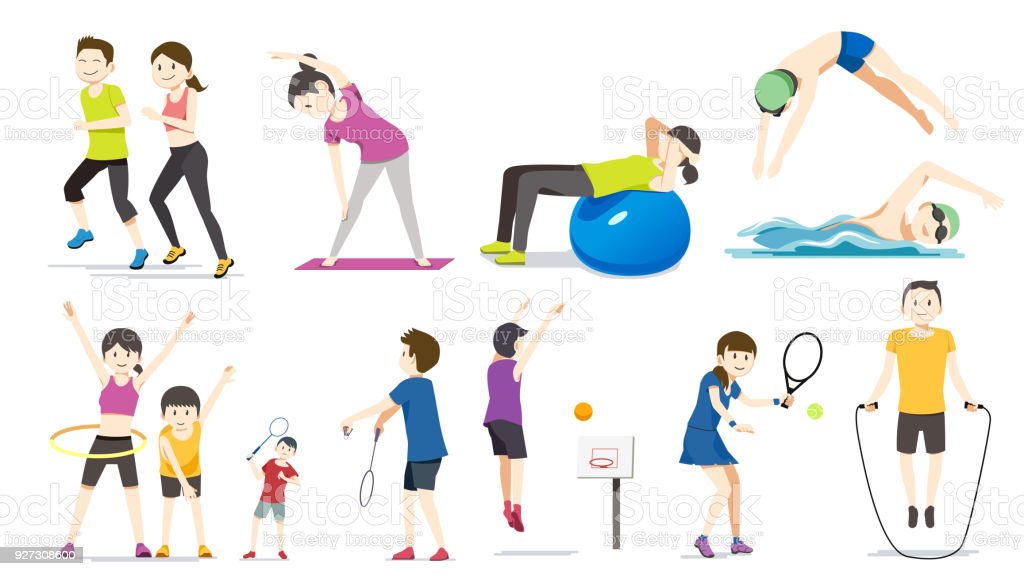


**Eat well**

- A healthy diet can help you manage cancer side effects, recover quicker, and improve health. It may also lower your future risk of cancer. Here are our tips to help you develop healthy eating habits:
- Include an assortment of veg­etables in every meal. Vegetables should be the centerpiece of your meal, not just a side dish.
- Eat foods high in fiber. These include whole grains, beans, peas, lentils, nuts, and seeds.
- Include probiotic and prebiotic foods to support a healthy gut. Probiotic foods include yogurt, kefir, sauerkraut or other fermented vegetables, miso, pickles, tempeh, kimchi, kombucha. Prebiotic foods are high-fiber foods and include chicory root, Jerusalem artichoke, dandelion greens, raw garlic, raw leeks, raw or cooked onion, raw jicama, and legumes and beans.


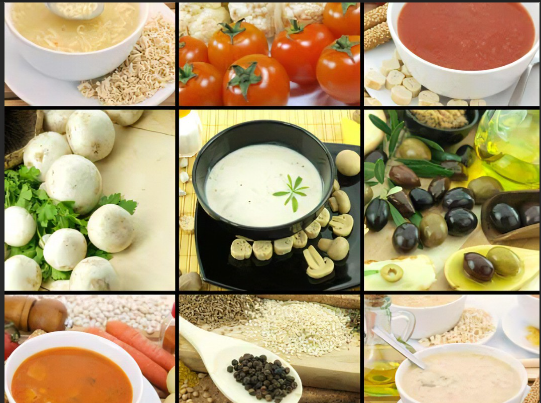


- Choose less red meat, like beef, pork, lamb, goat, veal, and bison, and more fish, poultry, and plant-based proteins, such as beans.
- Avoid processed meats, such as sandwich meats, burgers, chhoila, kachela.
- Include omega-3 and monounsaturated fats in your daily diet. Good sources include olive and canola oil, olives, walnuts, chia seeds, flaxseed, and avocado. Eat smaller portion sizes. An easy way to start is to use smaller plates and bowls when you eat.
